# Research Progress for Probiotics Regulating Intestinal Flora to Improve Functional Dyspepsia: A Review

**DOI:** 10.3390/foods13010151

**Published:** 2024-01-02

**Authors:** Xinyu Shen, Aijun Xie, Zijing Li, Chengxi Jiang, Jiaqi Wu, Mohan Li, Xiqing Yue

**Affiliations:** 1 College of Food Science, Shenyang Agricultural University, Shenyang 110866, China; shenxinyu98218@163.com (X.S.); 2022149037@stu.syau.edu.cn (Z.L.); 2022149044@stu.syau.edu.cn (C.J.); 2022240300@stu.syau.edu.cn (J.W.); 2 Department of Chemical and Biomolecular Engineering, National University of Singapore, Singapore 119077, Singapore; axie26@wisc.edu; 3 Shenyang Key Laboratory of Animal Product Processing, Shenyang Agricultural University, Shenyang 110866, China

**Keywords:** probiotics, gastrointestinal tract, intestinal flora, function dyspepsia

## Abstract

Functional dyspepsia (FD) is a common functional gastrointestinal disorder. The pathophysiology remains poorly understood; however, alterations in the small intestinal microbiome have been observed. Current treatments for FD with drugs are limited, and there are certain safety problems. A class of active probiotic bacteria can control gastrointestinal homeostasis, nutritional digestion and absorption, and the energy balance when taken in certain dosages. Probiotics play many roles in maintaining intestinal microecological balance, improving the intestinal barrier function, and regulating the immune response. The presence and composition of intestinal microorganisms play a vital role in the onset and progression of FD and serve as a critical factor for both regulation and potential intervention regarding the management of this condition. Thus, there are potential advantages to alleviating FD by regulating the intestinal flora using probiotics, targeting intestinal microorganisms. This review summarizes the research progress of probiotics regarding improving FD by regulating intestinal flora and provides a reference basis for probiotics to improve FD.

## 1. Introduction

Functional dyspepsia (FD) is a chronic non-organic gastrointestinal disease that is one of the most common diseases of the digestive system and one of the most common diseases of the gut–brain interaction worldwide [1,2]. Approximately 16% of individuals within the general population are affected by epigastric pain syndrome (EPS) and postprandial distress syndrome (PDS), which are two primary forms of FD [3,4]. In countries outside of Asia, the incidence is approximately 10–40%, and the incidence in Asian countries is approximately 5–30%. Of the people who experience FD, approximately 40% choose to seek medical treatment due to discomfort [5]. In approximately 80% of patients suffering from dyspepsia there is no structural explanation for their symptoms, which is then termed FD. The Rome IV criteria serve as the established diagnostic standard for FD. It is defined as a symptom of dyspepsia originating in the stomach and duodenum in the absence of evidence of organic, systemic, or metabolic disease that explains the symptom with its defining symptoms comprising postprandial satiety, epigastric pain, early satiety, or epigastric burning that persists for a minimum duration of six months [6,7]. As far as the pathophysiology of the disease is concerned, the Rome IV criteria as well as a recent multinational consensus of European experts support the role for impaired gastric accommodation, gastric distention hypersensitivity, disturbances in gastric emptying, and altered central nervous system signal processing [8]. The presence of lower gastrointestinal symptoms like diarrhea and constipation enhance the capacity of physicians to differentiate between individuals with functional digestive disorders and those experiencing non-FD [9,10]. FD is a chronic condition without a known cure, and as a result, it profoundly influences both the physical and mental health and the overall quality of life of patients [11].

The etiology and pathogenesis of FD remain unclear, but it is typically believed that it may be related to factors that include (1) gastric motility disturbance, (2) visceral hypersensitivity, (3) decreased gastric fundus receptivity diastolic function, (4) *Helicobacter pylori* infection, (5) gut–brain axis disturbance, (6) mental and social factors, and (7) increased eosinophilic cells, epithelial barrier disruption, and mucosal inflammation in the duodenum accompanied by elevated mast cell levels [12,13,14,15,16]. Additionally, an imbalance in intestinal flora is one of the important pathogenesis processes of FD. The imbalance in intestinal flora will lead to the disturbance of the intestinal environment, ultimately resulting in the reduction in the total amount of probiotics and a series of acute and chronic diseases [17,18]. Thus, the correlation between the gut microbiome and the development of human diseases is of paramount importance [19]. There are several lines of evidence suggesting that both locoregional duodenal and systemic changes may also be present in FD. Duodenal eosinophilia, epithelial barrier defect, and subtle mucosal inflammation, along with higher levels of mast cells, have been reported in FD, whereas the role of local and systemic inflammatory changes and increased small-bowel-homing T cells were not highlighted until very recently [20,21]. Intestinal flora may improve the clinical symptoms of FD by improving intestinal barrier function and visceral hypersensitivity, and regulating gastrointestinal motility. Changing the type and composition of intestinal flora may provide a safe and effective treatment to relieve FD symptoms [22].

**Figure 1:**
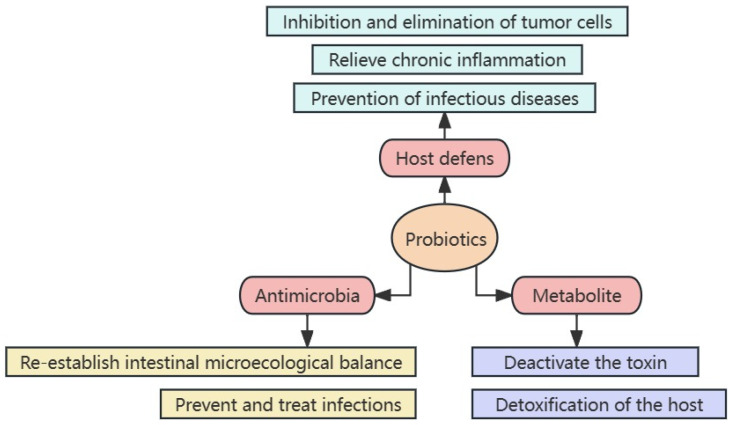
Mechanism of probiotic actions.

Probiotics are active microorganisms that are beneficial to the host. Furthermore, strains that can meet the basic conditions of probiotics can become probiotics. Therefore, probiotics cover numerous types of microorganisms, and the main probiotics in this study include *Bifidobacterium*, *Lactobacillus*, *Saccharomycetes*, *Bacillus*, and others [23,24]. Concurrently, the health effects of probiotics have also become a research hotspot (Table 1). Presently, probiotics have been demonstrated to maintain the normal structure of intestinal flora, resist pathogen infection, improve constipation and diarrhea, relieve lactose intolerance, reduce serum cholesterol levels, and promote immune system development [25]. The mechanism of action of probiotics primarily includes three aspects: (1) enhancing the host defense capacity, (2) directly fighting microorganisms, and (3) metabolites playing an important function [26] (Figure 1). Probiotics can effectively relieve functional gastrointestinal disorders such as irritable bowel syndrome [27]. Several intestinal flora colonize the gastrointestinal tract, an organ involved in the pathogenesis of FD due to mucosal damage and inflammation [15]. Therefore, improving FD symptoms by regulating the gut microbiota may be beneficial.

## 2. Relationship between Probiotics and Intestinal Flora

There is a close relationship between probiotics and gut flora. As a safe and effective intervention method, probiotics can affect the occurrence and development of various diseases by regulating the intestinal flora, and this also makes probiotics more attractive in the fields of basic and clinical research [49]. Humans acquire their initial gut flora from their mothers from birth, and the gut flora in infants is dominated by *Bifidobacterium*. With the establishment of human gut microbiota symbiosis, the gut microbiota changes with factors such as the environment, diet, and age, and the gut microbiota possesses a wide range of personalization [50]. Probiotics and intestinal flora interact through nutritional competition, antagonism, and symbiosis [51]. There is further proof that probiotics in this situation lessen the negative effects of antibiotic treatment, and certain probiotics possess the potential to completely eradicate *H. pylori* through an inhibitory impact on the bacterium. [52]. The direct regulation of probiotics on the intestinal flora depends upon the composition of individual flora, and the probiotic effects of different probiotic strains vary. Additionally, not all probiotic strains can exert beneficial effects on specific diseases [53].

### 2.1. Effects of Probiotics on Gastrointestinal Health

The notion of probiotics traces back to a hypothesis initially advanced by Russian researcher Elie Metchnikoff. He linked the extended lifespan of Bulgarian farmers to their consumption of fermented dairy products, and this idea was subsequently expanded upon [23,54,55,56]. In recent years, the research and product development of probiotics have both attracted greater attention. Probiotics can improve and regulate intestinal health, effectively relieve intestinal discomfort such as constipation, diarrhea, inflammatory gastrointestinal diseases, irritable bowel syndrome, and FD, and protect intestinal health to the greatest extent [57,58,59]. Through their colonization of the intestinal mucosa and interactions with the mucus layer, probiotics regulate the immune response, thus enhancing the ability to ward off external threats [60]. Following their establishment in the intestinal tract, the bioactive compounds responsible for their beneficial effects can help to maintain the equilibrium of the gut microbiota; this includes boosting the population of beneficial bacteria in the intestinal ecosystem while decreasing the abundance of harmful bacteria, ultimately bolstering intestinal immunity [61]. Changes in the composition of the intestinal flora can lead to gastrointestinal diseases, and probiotics can be applied to the intestinal environment to alleviate pathological conditions [51]. Bioactive molecules produced by probiotic strains, such as bacteriocins, vitamins, short-chain fatty acids, enzymes, and amino acids, exert certain beneficial effects on the host (Table 2). Studies have demonstrated that lactic acid bacteria can produce bioactive sequences of different compounds, such as peptides, sugar polymers, and fatty acids, that promote human health [62]. Hence, consuming fermented foods rich in probiotics can enhance the immune system and lower the risk of developing diseases. This occurs through continuous interaction between the bacteria and the host immune system, ultimately leading to alterations in the gut microbial composition that favor the growth of beneficial microbiota while regulating the presence of pathogenic flora [63,64].

### 2.2. Effect of Probiotics on Patients with FD

The pathogenesis of FD is complex. The strains of probiotics are specific; there are certain differences in the physiology and metabolism of the strains of different species, and the influence of different growth states of probiotics on intestinal flora and host metabolism is also different [74]. Studies have demonstrated that after the intervention of probiotics or their fermented products, the clinical symptoms in FD patients have been relieved to varying degrees, and their quality of life has been significantly improved (Table 3).

Wauters et al. [75] conducted a 16-week randomized, double-blind, placebo-controlled study of 68 FD subjects (as defined by Rome IV criteria) aged ≥18 years, in which 32 participants received probiotics and 36 received the placebo. The experimental group were provided with probiotic capsules (*Bacillus coagulans* MY01 and *Bacillus subtilis* MY02 in a 1:1 mixture with a bacterial count of 2.5 × 109 CFU) twice each day, and the placebo group were provided with maltodextrin capsules (without any symbiotic bacteria). The findings indicated a substantial increase in the quality of life and short-chain fatty acid (SCFAs) levels in the experimental group of FD patients (*p* < 0.05) coupled with a significant reduction in clinical symptom scores (*p* < 0.05). In contrast, the placebo group did not exhibit significant changes. The abundance of the intestinal flora *Rhodotella* and *Leuconostoidea* increased. The results revealed that *Bacillus coagulans* MY01 and *Bacillus subtilis* MY02 exerted some alleviating effects on FD. Navarro-Rodriguez et al. [76] conducted an 8-week randomized, double-blind, placebo-controlled clinical trial on 107 subjects diagnosed with *H. pylori* infection with FD. The results indicated that the bacterial eradication rate was 89.8% in the active probiotic group and 85.1% in the placebo group (*p* = 0.49). There was no significant change in the efficacy of bacterial eradication or the adverse effects of *H. pylori* eradication in the placebo and probiotic groups. This may be due to the low concentration of probiotics. Sun et al. [77] conducted a randomized clinical trial to determine the effect of beverages containing *Lactobacillus paracei* LC-37 and its ability to relieve FD symptoms. The results demonstrated that after 14 and 28 days of treatment with a beverage containing LC-37, the symptoms of 26 FD patients were relieved, clinical symptom scores were significantly reduced, abdominal pain and hiccups were significantly reduced after 14 days, and the symptoms almost completely disappeared after 28 days. There was a significant increase in *Lactobacillus*, *Lactococcus*, and *Weikerella*, while there was a significant decrease in the abundance of harmful bacteria such as *Bacillus folLiculiformis*. In general, the beverage containing LC-37 attenuates symptoms of FD, in part through strain-specific effects by increasing probiotics, such as *Lactobacillus* and *Weissella*, and decreasing harmful bacteria including *Lachnocliostridium*. Rahmani et al. [78] conducted a 4-week randomized, double-blind, placebo-controlled clinical trial in 125 FD patients according to Rome III criteria, in which 65 subjects received probiotics (*Lactobacillus reuteri*) and the rest received placebo. The results revealed that all FD-related variables, such as frequency, severity, and duration of pain, were significantly lower at the end of week 4 compared to the baseline. In this study, there was a relative success of probiotic-based treatment without significant improvement in recovery. Differences between the strains of bacteria used is a possible explanation of the discrepancies. In a study conducted by Nakea et al. [79], a comparison was made between the fundamental physiological properties of gastric fluid (GF) and the microflora structure within the GF among 44 healthy individuals and 44 FD patients. Subsequently, the FD patients were administered yogurt infused with the probiotic strain *Lactobacillus gasseri* OLL2716 (LG21 yogurt). The investigation assessed its impact on bacteriological parameters and symptoms while seeking to elucidate their interconnection. The results indicated that the volume of GF recovered in the stomach of FD patients after overnight fasting was greater than that of healthy subjects, while the volume of GF in the stomach of FD patients with improved symptoms after treatment with LG21 yogurt was reduced. Additionally, the overall structure of the bacterial community in the GF of FD patients and the abundance of *Prevotella* was significantly different from that of healthy subjects. In patients with FD, treatment with LG21 yogurt reversed this ecological imbalance. In a 12-week clinical trial, Ohtsu et al. [80] enrolled 116 FD patients with an average age of 42.8 ± 9.0 years, and all were Helicobacter-pylori-infected. The trial followed a double-blind, parallel, placebo-controlled, and randomized design. Participants were randomly allocated to either the group consuming yogurt enriched with *Lactobacillus gasseri* OLL2716 (the *Lactobacillus gasseri* OLL2716 group) or the group consuming yogurt without *Lactobacillus gasseri* OLL2716 (the placebo group). The findings indicated that *Lactobacillus gasseri* OLL2716 exerted a notably more favorable impact on gastrointestinal symptoms compared to that of the placebo group and exhibited statistical trends (*p* = 0.073). Specifically, the elimination rates of the primary FD symptoms were 17.3% for the placebo group and 35.3% for the *Lactobacillus gasseri* OLL2716 group (*p* = 0.048), thus underscoring the beneficial effect of *Lactobacillus gasseri* OLL2716 in regard to alleviating FD symptoms.

## 3. Relationship between Intestinal Flora and FD

Compared with healthy people, the species composition of the gut microbiota in FD patients showed significant changes. Intestinal flora may improve the clinical symptoms of FD by improving the intestinal barrier function, visceral hypersensitivity and regulating gastrointestinal motility.

### 3.1. Research Progress on FD

FD is often attributed to disorders of gastric physiology, such as slow gastric emptying, an inability to relax the gastric fundus after meals, or gastric hypersensitivity caused by gastric dilation [81]. The relaxation of the lower esophageal sphincter in gastroesophageal reflux is related to FD to some extent [82]. FD can also be induced by *Salmonella*, *Escherichia coli* O157, *Campylobacter jejuni*, *Giardia lamblia*, and norovirus [83]. FD can develop due to inflammation in the proximal small intestine or stomach following an intestinal infection, while irritable bowel syndrome (IBS) may be triggered by inflammation in the distal small intestine or colon. When inflammation affects both the proximal and distal segments of the small intestine, it can lead to a syndrome that combines features of both IBS and FD [84].

In clinical practice, drug therapy is still the main means of treating FD, including Helicobacter pylori eradication drugs, proton pump inhibitors (PPI), H2 receptor antagonists, antidepressants, and anti-anxiety drugs [85,86]. An analysis of 10 PPI randomized trials involving 3347 patients reported that histamine H2-receptor antagonists exerted a more pronounced effect than PPIs [87]. A PPI was effective in patients reporting regurgitant or ulcer-like FD but not in patients reporting dyspraxia like functional gastroenteritis [88]. For FD patients with abnormal gastric motility and fundus regulation, studies have demonstrated that motility-promoting drugs such as cisapride, domperidone, and itopride are more effective than placebo. They promote gastric motility and gastrointestinal peristalsis by stimulating smooth muscle contraction and 5-HT 4 receptors of neurotransmitter-regulating neurons. This is more suitable for patients with postprandial discomfort syndrome (PDS). Although the drug treatment exerts a certain effect, long-term use of drugs will cause diarrhea, dizziness, vomiting, rash, and other adverse reactions and may even be accompanied by headache, atrophic gastritis, stomach polyps, and other side effects [89,90,91]. In addition to drug treatment, there are placebo treatments and psychological treatments. A randomized clinical trial comparing the use of placebos to untreated IBS patients revealed that the use of placebos was significantly more likely to provide sufficient relief from symptoms [92]. For those who experience emotional disorders, psychological treatment can be considered. Although placebo treatment and psychotherapy resulted in fewer side effects, they provided less relief from FD symptoms [93]. Probiotics modulate intestinal dysbiosis and are in their infancy as a potential treatment for FD. Currently, the probiotics used in FD clinical trials include *Bifidobacterium bifidum* tablets, *Bacillus subtilis bifidum* capsules, *Clostridium butyricum* live bacteria capsules, and others [94,95]. Probiotics are considered to be a safe dietary supplement and offer potential benefits in managing the development and progression of symptoms in patients with IBS. This is potentially attributed to their anti-inflammatory effects or regulation of visceral hypersensitivity. Similarly (as with IBS), a mounting body of evidence suggests that addressing FD by implementing strategies aimed at restoring gastrointestinal flora represents a promising and evolving approach [96].

### 3.2. The Mechanism of Intestinal Flora on FD

#### 3.2.1. Regulation of Intestinal Barrier Function

Probiotics can regulate the intestinal flora, maintain the integrity of the intestinal barrier structure, and protect the intestinal tissue from damage by pathogens. The integrity of the intestinal barrier is a sign of intestinal health and a reflection of the ability of the microflora to adapt to the dynamic balance of mucosal function. The integrity of the mucus barrier is the first line of defense to protect the gastrointestinal tract, and the microbiome plays a major role in the factors affecting the mucus barrier by driving mucus changes [97]. The impaired intestinal barrier function caused by the imbalance in intestinal flora is associated with mild intestinal mucosal inflammation and immune activation [98]. FD patients exhibited lower mucin expression levels and higher mast cell (MC) counts compared with healthy individuals, indicating impaired mucosal integrity, i.e., increased intestinal permeability and mild inflammation [99]. FD patients with duodenal epithelial cells exhibited an abnormal expression of adhesion protein and increased paracellular channels, and there was a significant correlation between the expression level of intercellular adhesion protein and the degree of increased permeability and the severity of low-grade inflammation [100]. Intestinal microorganisms play an important role in maintaining the mucosal barrier function. Conversely, by synthesizing and secreting a variety of metabolites (such as SCFAs, indole derivatives, etc.), microorganisms promote the production and secretion of gastrointestinal mucus while increasing the expression of tight junction protein and cytoskeleton-related proteins. This plays a crucial role in regulating epithelial permeability [101,102]. Mediators such as histamine, rennet, and prostaglandin D2 released during MC activation regulate the secretion and permeability of chloride ions and water in epithelial cells. MC-mediated intestinal barrier changes are also associated with neuropeptides, neurotransmitters, hormones (vasoactive intestinal peptides, SP, NGF, estrogens, and estradiol), and inflammatory mediators (tumor necrosis factor-alpha, interferon-gamma, and cytokines) released by other immune cells [103]. Intestinal epithelial cell barrier function is impaired, ultimately resulting in the increased penetration of pathogenic bacteria and the activation of T lymphocytes to release inflammatory cytokines mediating inflammation and further aggravating the inflammatory response.

#### 3.2.2. Effects on Visceral Sensitivity

Visceral hypersensitivity indicates that the gastrointestinal mucosa of FD patients is strongly responsive to normal stimulation, and 35% to 50% of FD patients also have visceral hypersensitivity [104]. Visceral hypersensitivity is a crucial factor in functional gastrointestinal disorder syndrome, greatly influencing the occurrence and severity of symptoms. This is due to an abnormal mucosal immune response activation in relation to the level of inflammatory response and the close proximity of immune cells and sensory neurons [105]. There are numerous factors related to visceral hypersensitivity and signal transduction, such as the expression of cytokines, the secretion of corticosterone, the release of SCFAs, and microbial metabolites [106]. Research has revealed that a symbiotic intestinal microbiota is an essential requirement for the proper stimulation of intestinal sensory neurons, thereby offering a plausible mechanism for the transmission of information between the microbiota and the nervous system [107]. Increased visceral sensitivity can lead to abnormal pain or pain sensitivity in patients, and this is the main pathophysiological change in EPS. The sensitivity of the stomach and duodenum to mechanical and chemical stimulation was increased in patients with FD, and the degree of visceral sensitivity was consistent with the severity of clinical symptoms [108]. The hypersensitive state can be improved by downregulating the expression level of TRPV1 in the duodenum. TRPV1 can be selectively activated to induce the release of neuropeptides such as calcitonin gene-related peptide (CGRP), substance P, and others [109]. Evaluation of the physical injury response induced by inflammatory stimuli in both sterile and conventional mice suggests that certain symbiotic bacteria are necessary for the development of inflammatory hypersensitivity in mice. This suggests an important role of the interactions between symbiotic microbiota and the host in facilitating adaptation to environmental stress [110]. The intestinal microbiota significantly affects visceral hypersensitivity, and this is a new target for the treatment of FD-related visceral hypersensitivity.

#### 3.2.3. Regulating Gastrointestinal Motility

Gastrointestinal motility dysfunction is one of the main pathological mechanisms of FD. The generation of gastrointestinal motility is related to the slow-wave potential induced by interstitial cells of Cajal (ICCs). The change in the number or structure of ICCs is the key to gastrointestinal motility disorder, and the reduction in the c-kit gene may reflect the decrease in the number of ICCs [111]. It has been established that the decrease in plasma motilin content in FD patients is related to the decrease in gastric emptying capacity, gastric electrical rhythm disturbance, and antrum–pyloric–duodenal motor coordination disorder. Studies have reported that metabolites fermented by intestinal microorganisms such as short-chain fatty acids (SCFAs) or peptides, in addition to being an important energy source for gastrointestinal motility, can also directly activate the intestinal nervous system to regulate the synthesis and secretion of certain gastrointestinal hormones by intestinal endocrine cells. These include intestinal hormone peptide YY, cholecystokinin, glucagon-like peptide 1, gastric inhibitory peptide, and motion-related peptide, and they can also regulate gastrointestinal motility and gastric emptying [112,113]. Additionally, SCFAs stimulate the central nervous system (CNS) and enteric nervous system (ENS) to release the key neurotransmitter 5-HT, which can promote intestinal motor disorders through intestinal smooth muscle contraction and is one of the important factors affecting gastrointestinal motility [114]. Changes in the biosynthesis, release, or reuptake of 5-HT exert an important impact on the control of the central nervous system and gastrointestinal tract and are involved in the regulation of emotions, psychological states, and sensorimotor functions of the gastrointestinal tract [115,116]. The intestinal microbiota and its metabolites affect gastrointestinal peristalsis through various pathways, including intestinal neurons, glia, and intestinal macrophages [117]. During digestion, the complex migratory movement of the stomach and small intestine can mechanically migrate gastrointestinal contents and pathogenic bacteria to the distal intestine. Meanwhile, FD often exhibits non-transmissive and retrogressive MMC stage III activity that may induce or aggravate the migration of proximal intestinal bacteria to the stomach along with the regurgitant duodenal fluid. Delayed gastric emptying results in the retention of regurgitant bacteria in the stomach and duodenum for a longer period of time. Concurrently, the proliferation of foreign bacteria can produce lipopolysaccharides to stimulate the immune response and then inhibit gastrointestinal peristalsis, ultimately leading to the aggravation of FD symptoms [118,119]. Abnormal gastrointestinal motility may lead to a flora imbalance that, in turn, may further affect gastrointestinal motility and mediate the development of FD.

## 4. Mechanism of Probiotics to Improve FD Symptoms

Probiotics improve FD symptoms by regulating immune function and regulating the probiotics of metabolites such as short-chain fatty acids, bile acids, and neurotransmitters.

### 4.1. Regulation of Immune Function

**Figure 2:**
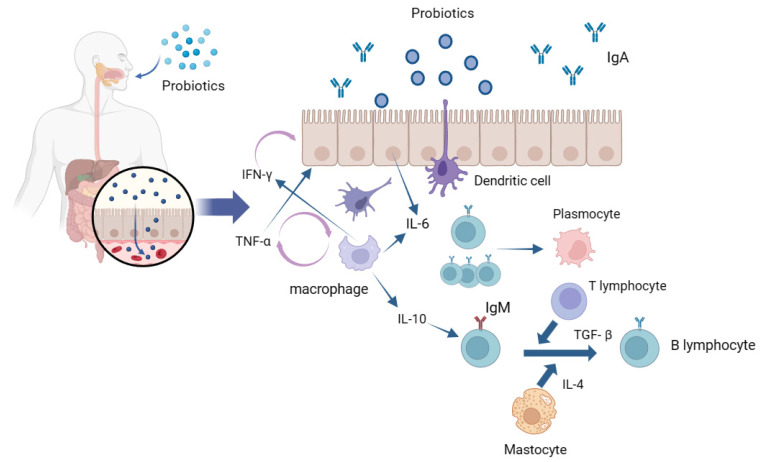
Local intestinal immune response induced by the interaction of probiotics and intestinal epithelial cells.

Probiotics exert a significant impact on shaping the gut microbiota composition, as they can hinder the establishment of pathogenic bacteria in the intestinal environment. They assist the host in developing a robust protective mucosal layer in the intestines, fortify the host’s immune system, and generate beneficial metabolites that contribute to maintaining overall health [120,121]. Local intestinal immune response is induced by the interaction of probiotics with intestinal epithelial cells and mucosal lamina propria immune cells (Figure 2). The gastrointestinal tract microbiota modulates the movement and role of neutrophils and influences the division of populations of T cells into various forms of T helper cells (Th), namely Th1, Th2, and Th17, or into regulatory T cells [122]. Mast cells (MCs) are membrane cells connected to the epithelial tissue; epithelial cells (ECs) and dendritic cells (DCs) are treated with probiotics and enter the intestinal lumen in different internalization manners. Following their interaction with epithelial cells, probiotics or their components are internalized and initially engage with antigen-presenting cells (APC), including macrophages and dendritic cells in the tunica propria of the digestive tract. The interaction between probiotics and epithelial cells triggers the release of IL-6 that, in turn, stimulates the clonal expansion of IgA-producing B lymphocytes, thereby elevating their population. These IgA-producing B lymphocytes then migrate into the protoplasmic cells of the intestinal tunica propria through these antigen-presenting cells [123,124]. Macrophages and dendritic cells engulf probiotics or fragments, thereby inducing the production of cytokines such as TNF-α and IFN-γ to enhance epithelial excitation and mutual interference among immune-related cells [125]. IL-6 and TNF-α are two important cytokines that play an important role in inflammation, anti-tumor activity, and the regulation of immune function. In the intestinal mucosal immune system, probiotic bacteria or their metabolites can be acquired and recognized by mucosal M cells as antigens. Thus, this promotes the development of the intestinal mucosal immune system, activating macrophages and B lymphocytes, forming germinal centers in the intestinal mucosal lymphoid tissue, and finally, transforming B lymphocytes into plasma cells to secrete mucosal antibody IgA to mediate mucosal immunity [126,127]. Probiotic strains can increase the levels of anti-inflammatory cytokines such as IL-10, reduce the levels of inflammatory cytokines such as TNF-α, IL-1β, and IL-8, and exert significant effects on reducing intestinal inflammation and improving colitis [128]. Additionally, different probiotics maintain gut health in different ways. *Lactobacillus* and *Bifidobacterium* primarily compete with pathogenic microorganisms for favorable adhesion sites, improve intestinal barrier function, regulate intestinal microflora, enhance intestinal immunity, and inhibit or kill harmful bacteria. However, *Bacillus* primarily enhances intestinal immunity through its metabolites and resists the invasion of pathogenic bacteria. Additionally, the probiotic effect of probiotics is strain-specific in regard to antibodies, and different strains utilize different modes of action and exert different effects on different parts of the intestine. For example, *Bacillus subtilis* MY02 is more effective at increasing SCFAs levels and *Lactobacillus paracei* LC-37 is more effective at reducing abdominal pain. Based on this, it is necessary to further precisely regulate the probiotics acting on different parts to provide full play to their probiotic effect on FD and other intestinal diseases [129,130].

### 4.2. Probiotics Work by Regulating the Metabolites of the Flora

**Figure 3:**
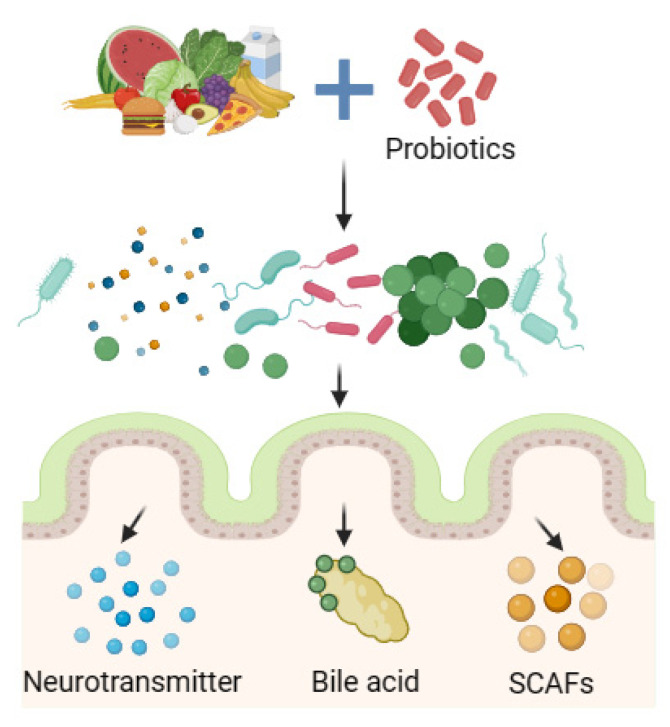
Probiotics regulate biota metabolites.

In addition to directly acting on the intestinal flora, probiotics can also play a beneficial role by indirectly regulating metabolites of the intestinal flora such as short-chain fatty acids (SCFAs), bile acids, and neurotransmitters, relieving FD symptoms to improve body health (Figure 3).

#### 4.2.1. Promotion of the Production of Short-Chain Fatty Acids

As significant byproducts of gut flora, SCFAs have the ability to strengthen the immunity and function as chemical messengers for brain–gut interactions [131,132]. Research has revealed that administering probiotics to mice led to a considerable increase in SCFAs and beneficial bacteria (including *Oscillibacter* and *Prevotella*) in their gut flora as compared to levels in the control group [133]. The gut is the primary site where SCFAs mediate intestinal epithelial integrity or mucosal immune response. Intestinal flora disturbances that lead to reduced SCFAs are associated with colon disease. SCFAs exert an anti-inflammatory function by regulating immune cell chemotaxis, reactive oxygen species release, and cytokine release [134]. Lactobacillus and *Bifidobacterium* produce lactic acid and acetic acid, which are major end products of carbohydrate metabolism. These organic acids, when produced in situ, can reduce intracavitary pH and inhibit the growth of pathogenic bacteria [135,136]. *Bifidobacterium* mainly produces SCFAs through fermentation, where the oligosaccharides of Chinese yams can be used as carbon sources by *Lactobacillus plantarum*, *Bifidobacterium*, and other intestinal probiotics in the simulated colon environment. The content of acetic acid in the fermentation broth of *Bifidobacterium* after 48 h was as high as 1.85 mg/mL, and after 8 h fermentation, was as high as 0.082 mg/mL [137]. SCFAs are produced by various pathways, where the most common is through glycolysis, and certain bacterial groups such as *Bifidobacterium* can also use the pentose phosphate pathway to produce the same metabolite [138].

#### 4.2.2. Promotion of Neurotransmitter Production

There is bidirectional regulation between the gastrointestinal tract, enteric nervous system, and central nervous system. Certain probiotic strains produce small molecules that exert different effects on the host and its gut microbes [139]. Neurotransmitters are important signaling molecules between neurons, and between neurons and effector cells and include dopamine, gamma-aminobutyric acid (GABA), and 5-HT [140]. Both dopamine and GABA are important neurotransmitters that regulate various functions in the central nervous system. Studies have demonstrated that a partially specific gut microbiota produces neurotransmitters such as dopamine and GABA. 5-HT, also known as serotonin, is an important metabolite of tryptophan, and the majority of 5-HT is produced by enterochromaffin cells that play an important role as an important neurotransmitter and signaling molecule in the two-way communication system between the brain and the gut [141,142]. The gut microbiota can promote the production of some neurotransmitters, which are often associated with central nervous system diseases such as the brain. Therefore, targeting the regulation of microbial metabolites, such as neurotransmitters, may be a potential way to improve neurological-system-related diseases. It has also been shown that *Candida*, *Streptococcus*, and *Enterococcus* can produce neurotransmitters such as serotonin; *Bacillus* and *Saccharomyces* species can produce noradrenaline; while *Lactobacillus* and *Bifidobacterium* species can synthesize and release GABA. These microbially synthesized neurotransmitters can act locally and also cross the intestinal mucosa to act locally, but potentially also the central nervous system via nerval signaling [143]. Through the regulation of neurotransmitters, the brain–gut axis can improve the symptoms of intestinal peristalsis, upper abdominal discomfort, loss of appetite, and constipation in FD patients [144].

#### 4.2.3. Promotion of Bile Acid Production

Under the action of gut bacteria, the primary bile acids formed in the liver are modified into secondary bile acids. The circulation process between the liver and the intestine is called enterohepatic circulation, which is also an important means to regulate the composition of bile acids [145,146]. Based on the important role of intestinal flora in the modification of bile acids, probiotics, as an intervention targeting intestinal flora, may indirectly regulate the metabolism of intestinal flora by bile acids, thereby playing a probiotic role in alleviating FD symptoms.

## 5. Probiotics as a Potential Treatment for FD

Interactions between the microbiota and host crosstalk are plausible underlying mechanisms, which will help to establish probiotics as a novel, tailored therapeutic approach for FD. Probiotics play a multifaceted role, and they contribute to the amelioration of FD symptoms through several mechanisms, such as eliminating pathogenic bacteria to reestablish microbial balance [98], modulating epithelial barrier permeability, influencing visceral hypersensitivity, exerting both local and systemic anti-inflammatory effects, and regulating intestinal motility [147,148]. These factors are very beneficial to intestinal health. Probiotics play a certain role in maintaining the integrity of the duodenal mucosa. FD is related to a defect in the duodenal barrier that is caused by the immune response of food and microorganisms in the local area and the whole body, thus producing FD symptoms [149]. *E. coli*/*Shigella* bacteria represent a significant origin of toxic lipopolysaccharides that may impede gastric emptying. The intake of probiotics, particularly *Bifidobacteria*, can efficiently lower their concentrations and reinstate normal motor function in the small intestine [150]. Patients with FD exhibit both local and systemic immune activation. Probiotics exert their influence by modulating toll-like receptors (specifically TLR2 and TLR4) and generating pro-inflammatory cytokines through the metabolites produced by the gut microbiota. The interaction between intestinal neurons and microorganisms increases neuronal survival and gastrointestinal motility. The TLR4 agonist lipopolysaccharide promotes the survival of intestinal neurons by activating TLR4 and NF-B. Factors that regulate neuronal TLR4 signaling may alter gastrointestinal motility [151]. Thus, TLR4 and its downstream signaling molecules could be potential therapeutic targets for the treatment of gastrointestinal motility disorders.

## 6. Conclusions and Future Trends

Probiotics, by regulating intestinal flora, offer a potential advantage in alleviating FD, addressing the disorder’s onset and progression through their roles in maintaining microecological balance, improving barrier function, and regulating the immune response. Currently, the diversity, structure, abundance, and distribution of FD-associated intestinal microbiota are abnormal. However, there is no unified conclusion as to which specific microbiota are closely related to the occurrence and development of FD. Regarding improving FD symptoms, there is a tendency for probiotics to exert beneficial effects, but there is still confusion regarding which strains or species may be beneficial or most beneficial. Furthermore, for all the randomized controlled trials that we identified, the longest treatment duration was limited to 16 weeks, thus leaving the long-term effectiveness of probiotics or prebiotics in FD undetermined. Variability in the clinical outcomes of probiotics for FD exists due to factors such as individual living conditions, medication use, and the underlying causes of the condition. Therefore, future research should place greater emphasis on examining the influence of probiotics in diverse populations and various FD subtypes. Moreover, the interaction between microbiota and host crosstalk is a possible underlying mechanism that may be useful to explore in order to further elucidate the mechanisms by which probiotics enhance FD.

## Figures and Tables

**Table 1:** The health effects of common probiotics.

Probiotic Species	Bacterial Strain	Mechanism of Action	Health Benefits	References
*Lactobacillus*	*Lactobacillus acidophilus*	Adjuvant antigen-specific immune response;	Immunity enhancement; lower inflammatory factors; restoration of nervous system function	[28,29]
	*Lactobacillus casei*	Through antagonism, colonization competition, increasing antibody production, enhancing the systemic immune effect, and the antibacterial action of metabolites, it can resist the invasion of pathogens	Inhibition of pathogenic bacteria in the gut; protecting the internal environment; maintaining intestinal microecology	[30,31]
	*Lactobacillus paracasei*	Regulating the production of anti-inflammatory cytokines by Th1/Th2 cells and reducing the release of toxic nitrogen-containing metabolites	Relieving inflammation; relieving chronic metabolic diseases	[32,33,34]
	*Lactobacillus rhamnosus*	Competitive colonization, inhibition of *H. pylori* growth, and adhesion to mucosal cells	Maintaining intestinal barrier integrity; inhibiting inflammation and oxidative stress; regulating gut–brain communication	[35]
	*Lactobacillus reuteri*	Increasing the number of *Bifidobacteria* in the gut, and transforming the intestinal dominant flora suitable for breaking down proteins into the flora suitable for sugar metabolism, thereby reducing the production of toxic and spoilage metabolites	Secreting antimicrobial compounds; regulating the host immune system; preventing diarrhea and colitis; reducing the prevalence of acute abdominal pain in infants	[36,37,38]
*Bifidobacterium*	*Bifidobacterium longum*	Increasing the intestinal flora and inhibiting pathogenic bacteria	Maintaining intestinal health in early life; promoting the establishment of intestinal microbiota	[39,40]
	*Bifidobacterium animals*	Increasing antibody production	Resisting foreign pathogenic microorganisms	[41]
	*Bifidobacterium infantis*	Preventing excessive intestinal immune responses	Regulating intestinal flora; anti-hepatic fibrosis; anti-infection	[42,43]
Others	*Bacillus coagulans*	Enhancing the specific defense of non-specific antigens	Pathogen suppression; improved immune ability and growth performance of the body; improved intestinal digestion and absorption of nutrients; improved utilization of mineral elements in the body	[44,45,46]
	*Streptococcus thermophilus*	Producing vitamins and cofactors; activating immune function; affecting bile salt concentration	Improved body immunity; improved lactose intolerance	[47,48]

**Table 2:** The health role of active substances in probiotics in the gut.

Biologically Active Compound	Probiotics	Mechanism of Action	Health Benefits in the Gut	References
Bacteriocins	*Lactococcus lactis*	Located in the cytoplasmic membrane region of receptor binding on bacterial surfaces	Inhibition of pathogenic bacteria in the gut; helps gastrointestinal bacteria to survive	[65,66]
Vitamins	*Lactobacillus reuteri* JCM1112; *Lactobacillus fermenti* CECT 5716	Promotes *Bifidobacteria* to produce B-complex vitamins and maintain the health of the host intestine	Promotes energy metabolism; reduces intestinal inflammation	[67,68]
SCFAs	*Butyricicoccus pullicaecorum*; *Bifdobacterium sps.*	Receptors maintain homeostasis in the host cells by controlling the energy utilization of the host	The energy source of the colon cells	[69]
Enzymes	*Lactobacillus sp.* G3_4_1TO2; *Lactobacillus fermentum* E-3	Amylases and peptidases produced by probiotic organisms play a role in biochemical reactions of the host metabolism	Scavenges free radicals; hydrolyzes *β-galactoside*	[70,71]
Amino acids	*Fusobacterium varium*; *Clostridium sps.*	Regulates carbohydrate and lipid metabolism and alters host physiology	Provides host with essential amino acids	[72,73]

**Table 3:** The effect of probiotics on functional dyspepsia.

Probiotics	Experimental Subject	Intervention Time	Primary Outcome	Reference
*Bacillus coagulans* MY01, *Bacillus subtilis* MY02	68 patients	16 weeks	Quality of life and SCFA content↑	Wauters et al. [75]
*Lactobacillus acidophilus*, *Lactobacillus rhamnosus*, *Bifidobacterium bifidum* and *Streptococcus faecium*	107 patients	8 weeks	H pylori and adverse reaction↓	Navarro-Rodriguez et al. [76]
*Lactobacillus paracei* LC-37	26 patients	14 days, 28 days	14 days: Abdominal pain and hiccups↓ 28 days: Abdominal pain and hiccups disappear, *Lactobacillus*, *Lactococcus*, and *Weikerella*↑, *Bacillus folliculiformis*↓	Sun et al. [77]
*Lactobacillus reuteri*	125 patients	4 weeks	The frequency, severity, and duration of pain↓	Rahmani et al. [78]
*Lactobacillus* OLL2716	44 healthy control participants, 44 patients	3 mouths	GF volume in stomach↓	Nakea et al. [79]
*Lactobacillus gasseri* OLL2716	116 patients	12 weeks	Rate of elimination of major symptoms↑	Ohtsu et al. [80]

## Data Availability

Data are contained within the article.
